# Severity of MASH activity by iron-corrected MRI predicts insulin resistance and cardiometabolic risk in type 2 diabetes

**DOI:** 10.1210/clinem/dgag042

**Published:** 2026-02-11

**Authors:** Andrea Ortiz Rocha, Srilaxmi Kalavalapalli, Eddison Godinez Leiva, Diana Barb, Fernando Bril, Stephen A Marangi, Helena B Thomaides-Brears, Jens T Rosenberg, Nathaly Cuervo-Pardo, Romina Lomonaco, Enrique Valdez Saenz, Anu Sharma, Kenneth Cusi

**Affiliations:** Division of Endocrinology, Diabetes and Metabolism, Department of Medicine, University of Florida at Gainesville, FL 32610, USA; Division of Endocrinology, Diabetes and Metabolism, Department of Medicine, University of Florida at Gainesville, FL 32610, USA; Division of Endocrinology, Diabetes and Metabolism, Department of Medicine, University of Florida at Gainesville, FL 32610, USA; Division of Endocrinology, Diabetes and Metabolism, Department of Medicine, University of Florida at Gainesville, FL 32610, USA; Division of Endocrinology, Diabetes and Metabolism, Department of Medicine, University of Alabama, Birmingham, AL 35294, USA; Division of Endocrinology, Diabetes and Metabolism, Department of Medicine, University of Florida at Gainesville, FL 32610, USA; Perspectum, Gemini One, 5520 John Smith Drive, Oxford, OX4 2LL, UK; Advanced Magnetic Resonance Imaging and Spectroscopy Facility, McKnight Brain Institute, University of Florida at Gainesville, FL 32610, USA; Division of Endocrinology, Diabetes and Metabolism, Department of Medicine, University of Florida at Gainesville, FL 32610, USA; Division of Endocrinology, Diabetes and Metabolism, Department of Medicine, University of Florida at Gainesville, FL 32610, USA; Division of Endocrinology, Diabetes and Metabolism, Department of Medicine, University of Florida at Gainesville, FL 32610, USA; Division of Endocrinology, Diabetes and Metabolism, Department of Medicine, University of Florida at Gainesville, FL 32610, USA; Division of Endocrinology, Diabetes and Metabolism, Department of Medicine, University of Florida at Gainesville, FL 32610, USA

**Keywords:** diabetes mellitus type 2, MASLD/NAFLD, cT1, imaging, liver fibrosis, MRE

## Abstract

**Context:**

Multiparametric magnetic resonance iron-corrected T1 mapping (cT1) may help identification and risk-stratification of steatohepatitis (MASH).

**Objective:**

In type 2 diabetes, metabolic dysfunction-associated steatotic liver disease frequently advances to steatohepatitis with significant fibrosis and increased risk of developing cirrhosis. cT1 allows MASH risk-stratification by measuring liver disease activity (fibro-inflammation) but its association with cardiometabolic risk factors that drive liver disease progression in T2D (insulin resistance, lipotoxicity, and metabolic syndrome) remains unclear.

**Methods:**

We recruited 109 participants with T2D from primary care settings who were classified into four groups according to cT1 and liver fat content (LiverMultiScan): (1) without steatosis; (2) steatosis only without MASH; (3) mild–moderate MASH (cT1 ≥ 800 to ≤875 ms); and (4) severe MASH (cT1 > 875 ms). We also measured insulin resistance (HOMA-IR), adipose tissue dysfunction (Adipo-IR, plasma adiponectin), and several noninvasive tests of MASH necroinflammation or fibrosis severity (NIS2+®, CK-18, FAST, and MRE).

**Results:**

Higher MASH disease activity (cT1) was associated with more severe features of the metabolic syndrome as well as increased insulin resistance (HOMA-IR) and adipose tissue dysfunction (higher Adipo-IR and decreased adiponectin levels; all *P* < .01). Elevated cT1 correlated with worse hepatic necroinflammation (FAST, NIS2+®, and CK-18) and more severe steatosis and fibrosis (all *P* < .001).

**Conclusion:**

In people with T2D, worse MASH disease activity (measured by cT1) is associated with unfavorable cardiometabolic risk factors that are known to drive liver disease progression. Use of cT1 in this population may help early identification of at-risk individuals who would benefit from earlier aggressive intervention in primary care.

**Why did we undertake this study?**

• We undertook this study to explore the relationship between fibro-inflammation in MASH diagnosed by magnetic resonance cT1 imaging with insulin resistance and cardiometabolic drivers of liver disease in “real-world” primary care, internal medicine, and endocrinology settings.

**What is the specific question(s) we wanted to answer?**

• Is the severity of MASH measured by cT1 associated with insulin resistance, adipose tissue dysfunction, and cardiometabolic risk factors in people with T2D?

**What did we find?**

• We found that worse liver disease activity in MASH (ie, fibro-inflammation by cT1) corresponded to a stepwise worsening of hepatic and adipose tissue insulin resistance, more severe features of the metabolic syndrome. Higher fibro-inflammation correlated with higher ALT, AST, FAST, NIS2+®, and CK18 as well as with surrogate measures of liver steatosis (MRI-PDFF) and fibrosis (magnetic resonance elastography [MRE]).

**What are the implications of our findings?**

• In people with T2D, more severe MASH disease activity measured by cT1 is associated with an unfavorable cardiometabolic risk profile (insulin resistance, lipotoxicity, and hyperglycemia) that are established drivers of liver disease progression. Use of cT1 may help identify individuals with T2D who are at a higher risk of disease progression that may benefit from early aggressive intervention.

About 70% of people with type 2 diabetes (T2D) have metabolic dysfunction-associated steatotic liver disease (MASLD) and are predisposed to develop its more severe form—metabolic dysfunction-associated steatohepatitis with clinically significant fibrosis (ie, fibrosis stage ≥F2 or “at-risk” MASH) which places them on a path to cirrhosis (1, 2). The metabolic pathways driving MASLD in T2D act synergistically to promote steatohepatitis and fibrosis leading to adverse hepatic and extrahepatic outcomes (3-5). Recent guidelines call for primary care physicians and diabetes healthcare teams to risk-stratify all people with T2D for clinically significant fibrosis (6-9).

Although liver biopsy remains the reference-standard for fibrosis staging and disease prognosis, procedure-related risks and high cost make it impractical for early diagnosis or disease monitoring in primary care or endocrinology settings (10). This has led to a noninvasive 2-tier screening approach (ie, FIB-4 +/− liver stiffness measurement (LSM) by vibration-controlled transient elastography or VCTE-LSM) (6-9). However, this strategy has several known shortcomings. Among them is its inferior performance in primary care (in particular, low sensitivity) compared to when used in hepatology settings where advanced liver disease is common. Consequent false positives increase healthcare costs from unneeded referrals to liver specialists and cause patient anxiety from diagnostic uncertainty. Also, the 2-tier screening approach focuses on advanced fibrosis and does not inform about the presence or severity of disease activity (ie, necroinflammation), which is of relevance to end-stage liver outcomes. This calls for exploring innovative approaches to improve early identification and establish who are at risk of being disease progressors, especially in high-risk disease states such as T2D. It should be highlighted that even in people with T2D about one third will never develop steatosis and another third only will have uncomplicated steatosis, making it a challenge to identify correctly those who have MASH and/or clinically significant fibrosis, or in other words those who are on a path to cirrhosis (11).

Cardiovascular disease is the main cause of mortality in people with T2D as well as in MASLD (12, 13). There is increasing evidence that cardiovascular disease affects those with MASH more compared to those with uncomplicated steatosis, even when accounting for established cardiometabolic risk factors (14-16). There is a strong association between MASH and nontraditional cardiovascular risk factors such as insulin resistance (IR) and adipose tissue dysfunction/lipotoxicity (17-19). This is clinically relevant not only from a cardiovascular but also a liver disease perspective because both traditional (ie, metabolic syndrome) and nontraditional risk factors combine to drive liver disease. However, we have limited information in people in the early stages of MASLD followed in primary care settings and our current 2-tier noninvasive screening approach (ie, FIB-4 +/− VCTE-LSM) cannot establish liver disease activity (fibro-inflammation). Greater access and shorter duration of testing have made MRI-based diagnostic approaches more clinically feasible. Multiparametric MRI, based on the use of quantitative MRI with liver iron content correction by T1 (cT1), quantifies changes to water in biological tissues arising from inflammation, edema, and/or fibrosis (20). It has been validated as a biomarker of disease activity and severity in MASH (21, 22) and other liver conditions. Used simultaneously with magnetic resonance proton density fat fraction (MRI-PDFF) for liver fat quantification (LiverMultiScan), cT1 has proved to be highly reproducible (23) and has been adopted to assess the prevalence of MASLD/MASH in the general population (24), for screening in research settings (22) and to monitor treatment response in MASLD (25).

Elevated cT1 have been reported to predict both liver and cardiovascular outcomes (26, 27). The role of liver cT1 as a complement to FIB-4 risk-stratification in early stages of MASLD, as seen in primary care or endocrinology clinical settings, remains to be fully explored but may help develop more cost-effective management strategies. Recent use of cT1 in adults with suspected MASLD in hepatology settings has proved to be cost-effective (28) and able to identify a significant number of patients with at risk of developing MASH that would have been missed by FIB-4 (29, 30).

In this proof-of-concept study, we explored the early stages and the full spectrum of MASLD in individuals recruited from family medicine, internal medicine, and endocrinology clinics. We explored the role of diagnosing liver fibro-inflammation in this real-world setting and the link between liver and cardiometabolic disease. We hypothesized that fibro-inflammatory disease activity by cT1 would be associated with an increased presence of cardiometabolic drivers of liver disease progression (ie, IR, lipotoxicity, hyperglycemia, and metabolic syndrome). If so, this proof-of-concept study is a first step toward understanding the potential role of cT1 in primary care for the diagnosis and management of people with T2D and MASH and to encourage larger, long-term prospective studies.

## Materials and methods

### Participants

A total of 117 participants (109 with T2D and 8 healthy controls without diabetes) were recruited from primary care (internal medicine and family medicine) and endocrinology clinics at the University of Florida (UF), Gainesville, Florida. Participants included were adults between 35 and 75 years old identified between September 2018 and February 2024. All underwent a medical history, physical examination and had blood drawn for routine chemistries.

Exclusion criteria were any liver disease other than MASLD (ie, hepatitis B or C, autoimmune hepatitis, hemochromatosis, drug-induced hepatitis, and others), history of alcohol abuse or significant alcohol intake (defined as ingestion of >21 standard drinks per week in men and >14 standard drinks per week in women over a 2-year period preceding evaluation; 1 drink = 14 g of pure alcohol), being on medications that could induce hepatic steatosis or hepatotoxicity (ie, estrogen, amiodarone, methotrexate, raloxifene, glucocorticoids, and others), use of vitamin E ≥ 800 IU/day or pioglitazone or any FDA-approved drug for MASH, metabolic surgery, type 1 diabetes or other forms of diabetes, pregnancy or lactation, history of cancer in the past 5 years prior to enrollment, presence of an implanted electronic medical device (ie, pacemaker, as transient elastography cannot be performed), history of clinically significant heart, pulmonary or renal disease, and body mass index (BMI) > 50 kg/m^2^. A GLP-1 receptor agonist was allowed if the participant had been on a stable dose for at least 6 months prior to enrollment. The study was approved by the UF Institutional Review Board, and all participants were legally competent to provide written informed consent.

### Study design

This was a cross-sectional study aimed at examining the clinical value of cT1 to assess liver disease severity in relation to the severity of IR and cardiometabolic risk of individuals across the spectrum of MASLD. After each subject provided informed consent, participants gave a detailed medical history and had an assessment of alcohol intake (AUDIT questionnaire). Routine blood chemistries were obtained after an overnight fast to rule out secondary causes of liver disease. In addition, we measured IR by HOMA-IR (primarily an indicator of hepatic IR). Adipose tissue dysfunction was assessed by measuring IR (Adipo-IR) and plasma adiponectin levels. All individuals underwent for magnetic resonance imaging (MRI). Liver imaging included assessment of fibro-inflammation by cT1, liver steatosis by MRI-proton density fat fraction (PDFF) and of liver fibrosis by magnetic resonance elastography (MRE). Liver steatosis and fibrosis were also assessed by VCTE. Metabolic syndrome was defined based on IDF criteria (31).

### Study measures

#### Iron-corrected T1 mapping (cT1), MRI-PDFF, and MRE

Iron-corrected T1 mapping (cT1) and MRI-PDFF were assessed using multiparametric MRI delivered via the FDA-approved LiverMultiScan platform/software (Perspectum, Oxford, UK) using a standardized MRI protocol at the University of Florida Advanced Magnetic Resonance Imaging and Spectroscopy (AMRIS) facility. All MRI scans were performed and certified by experienced radiologists and radiology technicians. The cT1 measurement, reported in milliseconds (ms), provided a quantitative assessment of liver fibro-inflammation, serving as an early indicator of disease progression, while MRI-PDFF, expressed as a percentage, quantified hepatic fat content, with high sensitivity and specificity for grading hepatic steatosis. For the purposes of MASLD and MASH stratification, participants with a cT1 value <800 ms and a PDFF <5.6% were classified as not having MASLD. Mild-to-moderate MASH was defined by a cT1 between 800 and 875 ms, and severe or high-risk MASH was defined as a cT1 > 875 ms, based on associations to extensive biopsy-paired and outcome data (22, 26, 27). MRE was also performed during the same MRI session to assess liver stiffness as a marker of fibrosis, with results expressed in kilopascals (kPa), correlating with fibrosis severity, and is predominantly effective in detecting advanced fibrosis and cirrhosis. MRE fibrosis staging was based on validated cutoffs (32) with values of ≥2.65 kPa indicating at least stage F1 fibrosis, ≥3.14 kPa stage F2 or higher, ≥3.53 kPa stage F3 or higher and ≥4.45 kPa stage F4 fibrosis. All imaging data were processed and analyzed according to manufacturer protocols, and quantitative results were used for disease stratification and subsequent statistical analyses.

#### Vibration-controlled transient elastography

VCTE by Fibroscan® (model 530 compact; Echosens, Paris, France) was performed to assess liver steatosis by controlled attenuation parameter (CAP, expressed in decibels per meter [dB/m]) and liver fibrosis by LSM (kilopascals [kPa]), as described before (1). Measurement of LSM was considered reliable only if IQR/med < 30% and success rate > 60% and using the same signals as the one used to measure liver stiffness. Individuals were considered to have steatosis based if CAP ≥274 dB/m (33). Presence of clinically significant fibrosis (≥F2) was defined as LSM ≥ 8.0 kPa (33).

#### Metabolic measurements and fibrosis diagnostic panel

We measured routine chemistries including a lipid profile, HbA_1c_ as well as several blood biomarkers including plasma cytokeratin-18 (CK-18; a biomarker of hepatocyte apoptosis/cell death), M30 Apoptosense ELISA (PEVIVA-Diapharma group Inc, Ohio, USA) (7); NIS2+®, a blood panel developed to identify at-risk MASH (MASH ≥ 4 with clinically significant fibrosis) (34) and plasma adiponectin levels were quantified using ELISA kit from Bio-techne, R&D Systems (Minnesota, USA) (4), an adipokine produced by adipose tissue that plays a critical role in the regulation of glucose and fat metabolism and a biomarker of healthy adipose tissue. Plasma FFA were measured using a colorimetric method from FUJIFULM Wako diagnostics (California, USA). Plasma insulin was measured using an ELISA (enzyme-linked immunosorbent assay) kit from ALPCO (New Hampshire, USA). We calculated HOMA-IR (fasting plasma glucose [mg/dL] × fasting plasma insulin [μU/mL])/405) and ADIPO-IR as fasting plasma FFA (mmol/L)×fasting plasma insulin (μU/mL), as previously reported (17-19).

For the evaluation of liver fibrosis stage, we used FIB-4 and FAST scores. FIB-4 was calculated as (age [years] × aspartate aminotransferase [U/L]/(platelet count [10^9^/L] × square root of alanine aminotransferase [U/L]). FIB-4 has been validated for fibrosis staging and prediction of future major adverse liver and cardiovascular outcomes (6-9). FAST was calculated as standardized from plasma AST, CAP, and VCTE-LSM (7).

### Statistical analysis

Data was summarized as mean ± SD or as percentages. For analysis, we divided individuals into 5 groups based on absence or presence of MR liver fibro-inflammation (cT1) and/or steatosis (MRI-PDFF): (1) healthy controls without obesity or T2D with a cT1 < 800 ms and MRI-PDFF <3.5%, (*n* = 8); (2) T2D without MASLD, defined as cT1 < 800 ms and MRI-PDFF <5.6% (*n* = 14); (3) T2D with MASLD, defined as cT1 < 800 ms and MRI-PDFF ≥5.6% (*n* = 11), (4) T2D with mild-to-moderate MASH defined as cT1 ≥ 800 to ≤875 ms (mild-to-moderate MASH; *n* = 31), (5) T2D with severe/high-risk MASH, defined as cT1 > 875 ms (severe MASH; *n* = 53). Comparisons between 3 or more groups were performed with ANOVA (with Bonferroni's adjustment for pairwise comparisons) and χ^2^ (or Fisher's exact test when appropriate). Comparisons between 2 groups were done with *t*-test and χ^2^ (or Fisher's exact test when appropriate). Partial Spearman correlations were performed between cT1 and individual cardiometabolic or liver disease biomarkers, adjusted for age and sex. A two-tailed value of *P* < .05 was considered to indicate statistical significance. Analyses were performed with Stata 15.1 (StataCorp LP, College Station, TX) and graphs with Prism 8.1.2 (GraphPad Software, Inc., La Jolla, CA).

## Results

### Clinical characteristics

The mean age of 109 participants with T2D was 57 ± 11 years; their BMI was 34 ± 6 kg/m^2^, AST and ALT were 30 ± 16 and 34 ± 23 U/L, respectively. The severity of MASH was associated with higher fasting plasma glucose, higher hemoglobin A1c, worse lipid profile, and higher plasma aminotransferases (ALT and AST) (Table 1). Sex, race and ethnicity did not have an impact for having MASH (*P* = .15 and *P* = .10, respectively). Individuals with severe MASH had a higher BMI compared to most other groups (all *P* < .001, except moderate MASH vs severe MASH, *P* = .225). More cardiometabolic risk factors were present among the groups with more advanced liver disease (Table S1) (35). People with MASH more often needed 2 or 3 glucose-lowering medications to control their diabetes. Metformin use was significantly higher in individuals with severe MASH (*P* < .001) and used most frequently compared with other glucose-lowering medications (Table S1) (35). Hypertension was present in more than half of the individuals in each group and with similar prevalence among the groups (*P* = .75; only 25% of healthy controls had hypertension; Table S1) (35). Atherogenic dyslipidemia was more prevalent among participants in the groups with MASH compared to the other groups (T2D without MASLD: 47%, MASLD: 73%, mild-to-moderate MASH: 74%, severe MASH: 85%; *P* = .02) but use of statins was similar among groups (*P* = .86) (Table S1) (35).

**Table 1:** Characteristics of patients with type 2 diabetes based on the presence of MASLD or MASH

*N* = 117	CONTROLS (without T2D or obesity) (*n* = 8)	T2D Without steatosis (*n* = 14)	T2D only steatosis (*n* = 11)	T2D with moderate MASH (*n* = 31)	T2D with severe MASH (*n* = 53)	*P* value (ANOVA)
Age (y)	46 ± 19	57 ± 14	57 ± 15	59 ± 10	57 ± 10	.10
Sex (male/female) (%)	50/50	64/36	36/64	58/42	74/26	.15
Race (%)						.10
Caucasian	88%	86%	64%	91%	89%	
African American	0%	14%	9%	6%	4%	
Asian	12%	0%	27%	0%	2%	
Other/not reported	0%	0%	0%	3%	5%	
Ethnicity (%)						.60
Hispanics	0%	0%	0%	10%	2%	
Not Hispanic	100%	100%	100%	90%	96%	
Other/not reported	0%	0%	0%	0%	2%	
BMI (kg/m^2^)	24.3 ± 3.1	29.3 ± 6.3	28.3 ± 4.1	35.0 ± 6.6	36.5 ± 5.2	<.001
BMI, %						<.001
Overweight	63%	57%	82%	16%	13%	
Obesity	0%	29%	18%	74%	87%	
Systolic blood pressure (mmHg)	124 ± 12	131 ± 19	131 ± 14	131 ± 14	131 ± 13	.71
Diastolic blood pressure (mmHg)	75 ± 9	71 ± 11	77 ± 9	78 ± 7	78 ± 8	.09
CAP (dB/m)	212 ± 35	259 ± 47	298 ± 48	328 ± 39	357 ± 32	<.001
≥274 (%)	13%	25%	55%	90%	100%	
≥288 (%)	0%	25%	45%	81%	98%	
VCTE-LSM (kPa)	4.8 ± 1.5	5.6 ± 2.7	5.1 ± 1.6	8.9 ± 4.6	10.4 ± 6.4	<.001
% with fibrosis ≥ F1	13%	17%	27%	71%	74%	
% with fibrosis ≥ F2	0%	17%	0%	42%	55%	
Liver disease activity (cT1, ms)	687 ± 53	727 ± 37	740 ± 31	839 ± 22	960 ± 71	<.001
Liver steatosis (MR-PDFF, %)	2.2 ± 0.7	2.9 ± 1.2	8.3 ± 2.6	11.0 ± 5.1	17.0 ± 6.8	<.001
FAST	0.07 ± 0.03	0.03 ± 0.03	0.09 ± 0.09	0.32 ± 0.17	0.47 ± 0.26	<.001
AGILE 3+	0.12 ± 0.09	0.34 ± 0.21	0.35 ± 0.20	0.56 ± 0.25	0.55 ± 0.27	<.001
NIS2+	0.23 ± 0.09	0.34 ± 0.23	0.36 ± 0.24	0.59 ± 0.23	0.66 ± 0.21	<.001
Laboratory data						
Fasting plasma glucose (mg/dL)	81 ± 7	105 ± 26	113 ± 33	113 ± 36	128 ± 49	.03
HbA1c (%)	5.1 ± 0.6	6.4 ± 1.1	6.6 ± 1.2	6.9 ± 1.0	7.2 ± 1.3	<.001
Total cholesterol (mg/dL)	192 ± 30	153 ± 38	169 ± 55	171 ± 46	164 ± 43	.34
Triglycerides (mg/dL)	93 (44-124)	110 (70-148)	144 (80-239)	139 (104-186)	154 (113-207)	.007
LDL cholesterol (mg/dL)	118 ± 30	78 ± 33	88 ± 47	92 ± 42	85 ± 37	.17
HDL cholesterol (mg/dL)	57 ± 13	54 ± 14	49 ± 11	49 ± 15	45 ± 11	.03
Non-HDL cholesterol (mg/dL)	136 ± 33	99 ± 35	119 ± 53	121 ± 43	119 ± 42	.37
Insulin resistance (HOMA-IR) (μU/mL × mg/dL)	1.4 ± 1.1	2.0 ± 2.0	2.9 ± 2.0	4.9 ± 1.0	7.5 ± 0.7	<.01
Adiponectin (μg/mL)	11.5 ± 5.9	4.9 ± 0.7	3.9 ± 2.0	4.1 ± 2.3	3.7 ± 2.6	<.001
Adipo-IR (mmol/L × μU/mL)	2.6 ± 2.4	2.3 ± 2.2	2.3 ± 0.5	6.4 ± 5.0	9.1 ± 4.9	<.01
ALT (units/L)	18 ± 8	16 ± 6	20 ± 9	30 ± 15	44 ± 27	<.001
Men >30 (%)	0%	0%	9%	23%	25%	.12
Women >19 U/L (%)	13%	7%	0%	39%	57%	<.001
AST (units/L)	22 ± 5	17 ± 4	20 ± 6	28 ± 9	37 ± 19	<.001
Men >30 (%)	0%	0%	9%	13%	17%	.36
Women >19 U/L (%)	50%	7%	0%	45%	58%	<.001
CK-18 (U/L)	138 ± 39	86 ± 35	106 ± 44	238 ± 188	335 ± 207	<.01
FIB-4 index	1.0 ± 0.5	1.0 ± 0.3	1.2 ± 0.5	1.5 ± 0.7	1.3 ± 0.6	.03
% with elevated FIB-4*^a^*	13%	0%	18%	45%	38%	.02

Values expressed as mean +/− SD.

Abbreviations: Adipo-IR, adipose tissue insulin resistance; ALT, alanine aminotransferase; AST, aspartate aminotransferase; BMI, body mass index; CAP, controlled attenuation parameter; CK-18, cytokeratin 18; FAST, FibroScan®-associated steatosis targeted; FIB-4, fibrosis-4 index; HDL, high-density lipoprotein, HOMA-IR, homeostatic model assessment for insulin resistance; LDL, low density lipoprotein; LSM, liver stiffness measurement; MASH, metabolic dysfunction-associated steatohepatitis; MASLD, metabolic dysfunction-associated steatotic liver disease; T2D, type 2 diabetes mellitus; VCTE, vibrant-controlled transient elastography.

^*a*^Elevated FIB-4 defined as ≥1.3 in participants <65 years of age (%) and ≥ 2.0 in participants ≥65 years old (%).

### Association of liver fibro-inflammation to cardiometabolic risk

**Figure 1:**
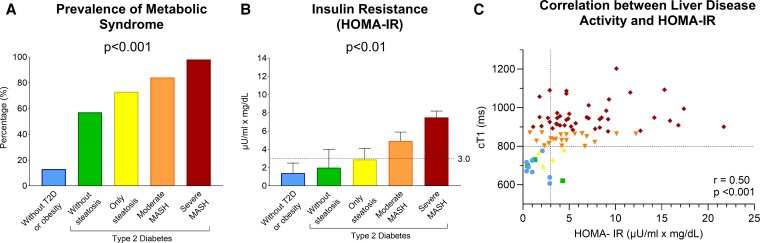
Prevalence of metabolic syndrome among study groups (A). Mean IR determined by HOMA-IR among the study. HOMA-IR = (fasting plasma glucose × fasting plasma insulin)/405 (in µU/L × mg/dL) (B). Correlation between magnetic resonance iron-corrected T1 (cT1) and HOMA-IR (C). Values expressed as mean ± SD. *P* value represents the analysis of variance for the entire cohort.

Metabolic syndrome was present in most individuals (84%), being highest in those with MASH (98%) compared to people with T2D but without MASLD (57%) or with uncomplicated liver steatosis (73%) (*P* < .001) (Fig. 1A). IR by HOMA-IR worsened with disease progression being worse in those with severe MASH (*P* < .01) (Fig. 1B). There was a significant correlation between liver fibro-inflammation (cT1) and more severe IR (HOMA-IR) (*r* = 0.50, 95% CI: 0.31-0.66, *P* < .001) (Fig. 1C). The marker of apoptosis CK-18 increased with MASH severity (mild-to-moderate MASH: 238 ± 188 vs severe MASH: 335 ± 207 U/L; *P* < .01) and correlated with the severity of fibro-inflammation (*r* = 0.43, *P* < .001). Higher cT1 was associated with higher BMI, higher plasma triglycerides and lower HDL-C (all *P* < .05).

**Figure 2:**
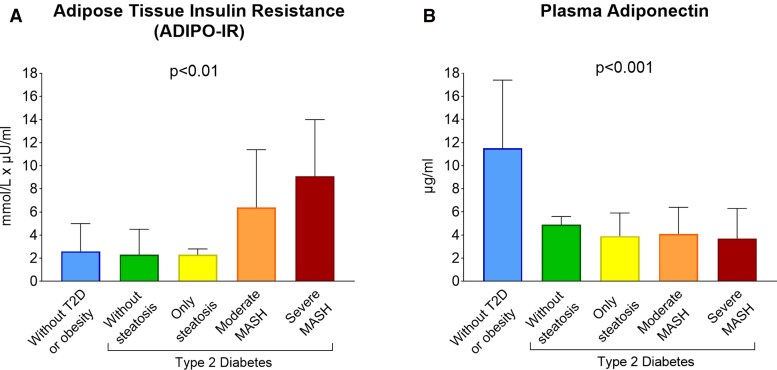
Mean adipose tissue IR index (Adipo-IR = fasting plasma FFA × fasting plasma insulin) among groups (A). Mean plasma adiponectin concentration in study groups (B). *P* value represents the analysis of variance for the entire cohort.

Adipose tissue dysfunction, as measured by Adipo-IR, was more pronounced with the progression of liver disease, ranging from 2.3 ± 0.5 with isolated steatosis, to 6.4 ± 5.0 with mild-to-moderate MASH, and 9.1 ± 4.9 in the group with severe MASH (*P* < .01) (Fig. 2A). Also, adipose tissue dysfunction/IR was evident from the lower plasma adiponectin levels (C: 11.5 ± 5.9 μg/mL, 4.9 ± 0.7 µg/mL in those with T2D but without steatosis, 3.9 ± 2.0 µg/mL in those with only steatosis, 4.1 ± 2.3 µg/mL in mild-to-moderate MASH and 3.7 ± 2.6 µg/mL in severe MASH; *P* < .001 for the trend) (Fig. 2B). Consistent with this, adiponectin correlated with the severity of fibro-inflammation (*r* = −0.41, 95% CI: −0.58 and −0.23, *P* < .001).

### Association of liver fibro-inflammation to liver disease activity

**Figure 3:**
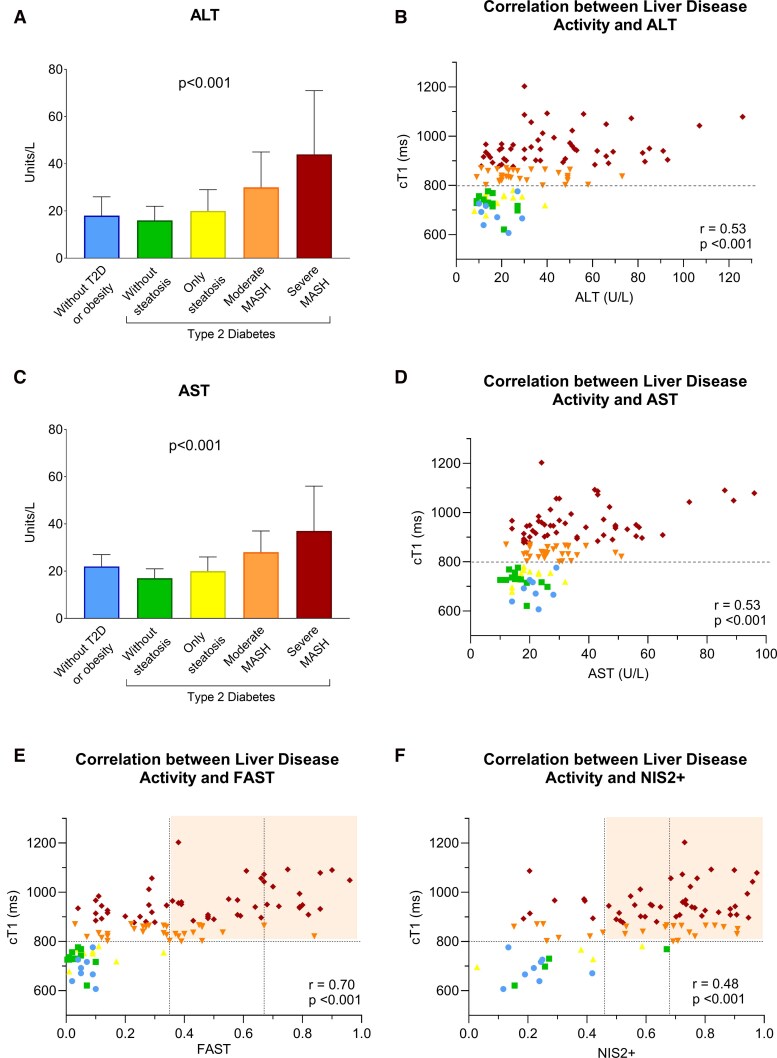
Mean plasma alanine aminotransferase levels (ALT) among groups (A). Correlation between ALT and magnetic resonance iron-corrected T1 (cT1) (B). Mean plasma aspartate aminotransferase (AST) among groups (C). Correlation between magnetic resonance iron-corrected T1 (cT1) and AST (D). Correlation between iron-corrected T1 (cT1) and FAST score (E). Correlation between magnetic resonance iron-corrected T1 (cT1) and NIS2+® (F). Values expressed as mean ± SD. *P* value represents the analysis of variance for the entire cohort.

Elevated cT1 correlated with other biomarkers indicative of liver disease activity in MASH (Fig. 3). Plasma aminotransferases were higher in individuals with severe MASH compared to all other groups, with abnormal values most prevalent in females (Table 1). Participants with severe MASH had the highest levels of plasma ALT compared to mild-to-moderate MASH and uncomplicated steatosis (44 ± 27 U/L vs 30 ± 15 U/L vs 20 ± 9 U/L, respectively, *P* < .001) (Fig. 3A). There was a robust correlation between ALT and cT1 (*r* = 0.53; 95% CI: 0.37-0.64, *P* < .001) (Fig. 3B) and AST with cT1 (*r* = 0.53, 95% CI: 0.37-0.65, *P* < .001) (Fig. 3D). Correlations with cT1 were also observed for the FAST score (Fig. 3E) (*r* = 0.70, 95% CI: 0.58-0.80, *P* < .001) and steatohepatitis biomarker NIS2+® (*r* = 0.48, 95% CI: 0.30-0.66, *P* < .001) (Fig. 3F). Other noninvasive scores and biomarkers were also correlated with cT1 (FIB-4: *r* = 0.20, *P* = .034; CK-18: *r* = 0.43, *P* < .001; and AGILE3+: *r* = 0.38, *P* < .001).

Participants with more severe MASH had a much higher liver fat content by MRI-PDFF compared to mild-to-moderate disease and uncomplicated steatosis (17.0 ± 6.8% vs 11.0 ± 5.1% vs 8.3 ± 2.6%, respectively, *P* < .001), as well as when measured by VCTE-CAP (Table 1). Hepatic steatosis measured by both MRI-PDFF and VCTE-CAP correlated with cT1 (*r* = 0.75, 95% CI: 0.66-0.81 and *r* = 0.68, 95% CI: 0.56-0.77; *P* < .001, respectively).

### Association of liver fibro-inflammation to liver fibrosis

Significant fibrosis by MRE was evident in those with mild-to-moderate and severe MASH with ∼40% in both groups having stage 2 fibrosis (mean MRE: 3.2 ± 1.5 and 3.1 ± 1.0 kPa, respectively, *P* = .01 within the entire cohort). Fibro-inflammation correlated with the degree of fibrosis by MRE (*r* = 0.34, 95% CI: 0.19-0.50, *P* < .001). More participants with severe MASH by cT1 had clinically significant fibrosis (stage ≥ F2) by VCTE-LSM compared to those with mild-to-moderate MASH (55% vs 42%, respectively, *P* < .01). The correlation between cT1 and VCTE-LSM was *r* = 0.50 (95% CI: 0.32-0.62, *P* < .001).

## Discussion

This proof-of-concept study is the first to examine, in people with T2D recruited from primary care and endocrinology clinics, the close relationship between liver disease activity in MASLD (ranging from early to advanced stages stratified by cT1) with IR and cardiometabolic risk. We found that worsening hepatic fibro-inflammation was associated with a stepwise increase in hepatic and adipose tissue IR (ie, dysfunctional adipose tissue) and features of the metabolic syndrome. Results were confirmed by the significant correlations between cT1 and ALT, AST, NIS2+™ (Fig. 3), and CK-18, as well as with liver imaging (FAST and MRE). This highlights a MASLD-cardiometabolic connection often overlooked by primary care and endocrinology clinicians. Thus, in selected individuals with T2D who are at the highest risk of MASH and cardiovascular disease, a diagnosis of fibro-inflammation by cT1 could be helpful when traditional risk-stratification (ie, by FIB-4 +/− VCTE-LSM) is equivocal (8, 9). Also, during treatment with glucose-lowering medications such as GLP-1RA or pioglitazone, normalization of plasma aminotransferases may suggest low-risk (FIB-4 < 1.3) although the presence of persistent fibro-inflammation may still lead to liver disease progression and negative cardiovascular outcomes (27). In summary, within our rapidly expanding noninvasive tests for MASH, cT1 may play a growing role in the risk-stratification of people with T2D.

Multiorgan T1-mapping has identified systemic patterns of liver, cardiac, pancreatic, and brain fibro-inflammation that are associated with subclinical organ dysfunction, morbidity, and increased mortality (36, 37). For instance, cardiac T1 can identify early stages of heart failure and cardiovascular disease beyond traditional risk factors. A recent study from the UK Biobank that included ∼33 000 participants reported that liver disease activity by cT1, but not liver fat (MRI-PDFF), was associated with higher risk of cardiovascular events, atrial fibrillation, heart failure, and all-cause mortality (26). The use of imaging modalities in MASLD such as cT1, MRI-PDFF, and MRE are increasingly essential for risk-stratification, diagnosis and monitoring disease activity in MASH, offering noninvasive, quantitative, and reproducible assessment of liver pathology. The relevance of chronic fibro-inflammation as a surrogate for increased cardiovascular risk may be mediated through nontraditional risk factors that have not been carefully assessed until recently (31). Among these, this study clearly establishes a robust link between fibro-inflammation and IR (Figs. 1B, 1C and 2A). Participants with the most severe liver fibro-inflammation by cT1 had the worse hepatic (HOMA-IR) and adipose tissue (Adipo-IR) insulin resistance. Therefore, liver fibro-inflammation by cT1 may become a valuable surrogate not only of liver disease activity but of overall metabolic health. This observation had been reported earlier in people with T2D who had undergone a liver biopsy to assess liver histology (17-19) but could not be translated into practical management in primary care or endocrine settings given the high costs and invasive nature of a liver biopsy. However, with cT1 being a potential cost-effective tool in primary care (28) and validated as a predictor of liver (21, 22) and cardiovascular (26) disease, it may now be possible to incorporate assessment of fibro-inflammation by cT1 earlier in people at risk of MASLD as it is being done in hepatology care already (28-30). Future work should evaluate how to best incorporate cT1 into risk-stratification strategies in primary care and endocrinology settings.

Adipose tissue dysfunction and lipotoxicity have been well established as risk factors in the development of MASH (3-5). In this proof-of-concept study people with steatohepatitis had a ∼3- to 4-fold increase in Adipo-IR and a ∼70% reduction in plasma adiponectin levels. We found an abrupt reduction in plasma adiponectin by the presence of diabetes, independent of having steatosis only or steatohepatitis (Fig. 2B). The steep and early reduction in adiponectin across groups with T2D, ranging from steatosis alone to severe MASH, signals perhaps that its decrease is an early signal of adipose tissue dysfunction and overall metabolic stress. This is also consistent with our prior work (17-19) but expands our understanding by showing that adiponectin is an earlier biomarker of metabolic dysfunction in MASLD than the rise of IR measured by Adipo-IR or HOMA-IR. This has also been suggested in large epidemiological studies where higher plasma adiponectin levels are associated with better metabolic health and its decrease with a transition to a metabolically unhealthy status (38) and with the development of T2D (39). In contrast, adipose tissue IR measured (Adipo-IR) appeared to be a more selective indicator of fibro-inflammation as it increased especially in those with steatohepatitis (Fig. 2B). This is consistent with studies where a higher Adipo-IR was a strong predictor of significant fibrosis (beyond HOMA-IR) in people with T2D (19). Moreover, increases in adiponectin by thiazolidinediones mediate histological improvement in MASH (40).

Current guidelines based on FIB-4 +/− VCTE-LSM focus on fibrosis stages (6-9). This has several advantages (simple, low-cost, easily adoptable in settings with limited resources, reasonable specificity, and backed by liver outcomes data). However, given the FIB-4 origin from staging viral hepatitis to current practice in MASLD it has some limitations that call for exploring complementary approaches. FIB-4 was developed for detection of advanced liver fibrosis stages (F3-F4) and has low sensitivity for F2 fibrosis stage and may miss many high-risk individuals (29). Even when VCTE-LSM is performed, being less accurate in earlier disease stages is a challenge. With the use of GLP-1RA becoming increasingly common in T2D, many patients will have normal or near-normal ALT and AST leading to a misleading low FIB-4 while there is still significant steatohepatitis. Finally, measuring the severity of MASH disease activity may potentially enhance risk-stratification of people in need of additional lifestyle or pharmacological treatments to prevent liver or cardiovascular disease (26, 27, 29). Recent studies suggest that cT1 may be the most cost-effective strategy to manage steatohepatitis (41) and help identify multiorgan abnormalities in people with T2D (42).

The study has some limitations. This study only examined the relationship between liver disease activity, IR, and cardiometabolic risk factors and not major liver or cardiovascular outcomes. However, cT1 has been reported to be an accurate and reproducible noninvasive test (23) and validated against liver histology as a predictor of future liver outcomes (21, 22) as well as for cardiovascular events (26) in large population studies. Future work should evaluate the integration of cT1 in the current MASLD/MASH screening strategies in T2D and its real-world utility. Also, future work should investigate the health economic benefit (including cost-effectiveness) of using cT1 in larger cohorts to detect MASH early and prevent both liver and cardiac outcomes.

In summary, our proof-of-concept study shows that cT1 is a robust marker of liver fibro-inflammation in primary care and endocrinology settings, having a strong correlation with both cardiometabolic risk factors and hepatic/adipose tissue IR. In clinical practice, beyond standard FIB-4 +/− VCTE-LSM risk-stratification, cT1 may help identify a subset of individuals with more severe IR and liver disease activity at greater risk for liver disease progression and cardiometabolic disease. Additional studies are needed to more fully establish the role of cT1 in clinical practice.

## Data Availability

Restrictions apply to the availability of some or all data generated or analyzed during this study to preserve patient confidentiality or because they were used under license. The corresponding author will on request detail the restrictions and any conditions under which access to some data may be provided.
